# The Effect of Executive Function on Word Recognition: Comparison Between Native Chinese and Learners with Chinese as A Second Language (CSL)

**DOI:** 10.1007/s10936-024-10120-6

**Published:** 2025-01-10

**Authors:** Cai Mingjia, Liao Xian

**Affiliations:** Department of Chinese Language Studies, Centre for Research on Chinese Language and Education, The Education University of Hong Kong, Tai Po, N.T Hong Kong

**Keywords:** Word recognition, Executive functions, Orthographic awareness, Morphological awareness, Chinese as a second language, Mediating effects

## Abstract

Word recognition is a fundamental reading skill that relies on various linguistic and cognitive abilities. While executive functions (EF) have gained attention for their importance in developing literacy skills, their interaction with domain-specific skills in facilitating reading among different learner groups remains understudied. This study examines the relationship between EF, orthographic awareness, morphological awareness, and Chinese word recognition in 204 Chinese as a second language (CSL) students and 419 native Chinese primary students. Our findings reveal that EF indirectly influences word recognition through orthographic awareness, but not through morphological awareness. The result of group comparison indicates that direct and indirect effects of EF on word recognition are evident in both native and CSL groups. Nevertheless, EF plays a more prominent role in CSL learners. These results contribute to our understanding of cognitive skills in reading and offer significant implications for instructional practices.

## Introduction

Word recognition serves as a crucial foundation for acquiring literacy and achieving academic success (Cai & Liao, 2024; Ober et al., 2019; Perfetti, 2007). However, it remains a complex and demanding process for young readers who are still developing their decoding skills (Perfetti, 2007; Spencer & Cutting, 2021). Word recognition necessitates the integration of written input with orthographic, phonological, and semantic representations stored in the mental lexicon, requiring the retrieval and integration of relevant knowledge (Seidenberg et al., 2011; Zou et al., 2022). Consequently, extensive research has been dedicated to investigating the underlying mechanisms of word recognition, identifying significant contributors such as orthographic awareness (Chan et al., 2021; Liu & Chung, 2022; Tong et al., 2019) and morphological awareness (Choi et al., 2018; Koda, 2021). More recently, there has been a growing focus on the role of executive functions (EF) in language learning (Gustmann & Poarch, 2022; Grant et al., 2015; Luque & Morgan-Short, 2021). EF has also been recognized as playing a prominent role in word recognition (Burgoyne et al., 2019; Chung & McBride-Chang, 2011; Fong & Ho, 2022; Raudszus et al., 2019).

Recent research indicates that the impact of both domain-general skills (e.g., EF) and domain-specific skills (e.g., linguistic skills like orthographic awareness and morphological awareness) on reading can vary significantly between native and non-native learners (e.g., Bialystok et al., 2010; Brothers et al., 2021). Chinese is a logographic and morpho-syllabic language that presents a significant contrast to alphabetic languages. It is less clear whether such characteristics could affect EF’s role among different groups of Chinese learners (Peng et al., 2018). Moreover, the number of CSL learners has witnessed a significant increase in recent decades (e.g., Gong et al., 2020), while many CSL learners encounter difficulties in the initial stages of learning due to the intricate nature of spoken and written Chinese (Gao et al., 2020; Zhao et al., 2013; Liao et al., 2022). It is thus necessary to unpack the relationship between EF and word recognition among native Chinese learners and CSL learners. This would not only enhance our understanding on the unique factors influencing word recognition in CSL learners but also facilitate the development of tailored instructional approaches (Gao & Gube, 2020; Ma et al., 2017). In a nutshell, this study could contribute to existing theories of literacy acquisition in both first language (L1) and second language (L2) learners.

## Literature Review

### Word Recognition in Chinese

The basic units of written Chinese are square-like characters composed of interwoven strokes, which starkly differ from those found in alphabetic languages (Chung & McBride-Chang, 2011). The number of Chinese characters is large. According to Zhang et al. (2017), there are over 7,000 characters and around 3,500 of them are common characters. Most characters consist of two radicals: semantic radicals, which provide semantic clues (e.g., 木, semantic radical, *wood*, in 林 /lam4/, *forest*), and phonetic radicals, which offer phonological cues (e.g., 青, phonetic radical, /cing1/, in 清 /cing1/, *clear*). Unlike alphabetic languages that tend to have consistent grapheme-phoneme correspondence, the reliability of phonetic and semantic radicals in Chinese is less predictable. For instance, it is estimated that fewer than 30% of characters maintain complete consistency with their phonological radicals (e.g., the phonetic radical 工 /gung1/ in the character 江 /gong1/, *river*) (Chung & Leung, 2018). Moreover, the role of certain radicals may vary across characters. For example, while the radical 馬 /maa5, *horse*/ provides phonological clues in the character 媽 /maa1/, *mother*, it serves as a semantic radical in the character 馳 /ci4/, *running like a horse*. The complex functions of radicals hinder the form-sound correspondence of Chinese characters, thereby increasing the challenge of Chinese word recognition (Ho et al., 2003).

Chinese is also known as a morpho-graphic language, wherein each basic written unit (i.e., character) represents a morpheme (Tong et al., 2017). Some characters can function as standalone words (i.e., single-character words), for example, 山 /saan1/, *mountain* and 水 /seoi2/, *water*. However, there is a significant presence of homonyms among single-character words in Chinese. For instance, the character 中 can refer to either /zung1/, *middle* or /zung3/, *win something*. The large number of homonyms may lead to confusion in matching the character with the appropriate morpheme, making it challenging to use morphological knowledge during word recognition in Chinese. Moreover, over 80% of words in Chinese are compound words, primarily composed of two characters (morphemes) (Tong et al., 2014). Due to the limited number of morphemes, morphemes are recursively used to form compound words, which lead to many compounding words embedding the same morpheme. Additionally, it is worth noting that while some words can be recognized by integrating the knowledge of their component morphemes (e.g., 書架 *bookshelf* can be deciphered by combining 書 /syu1/, *book* and 架 /gaa3/, *shelf*), this approach is not applicable to all words. For example, the word 東西 *stuff* is unrelated to either 東 /dung1/, *east* or 西 /sai1/, *west*. Given the complexity of Chinese orthography and word system, acquiring skilled word recognition in Chinese necessitates many years of parental and school-based instruction for learners to master the fundamental skills of word recognition (Chung & McBride-Chang, 2011; Li & Rao, 2000).

### Effects of Executive Functions on Word Recognition among L1 and L2 Learners

The executive functions (EF), which governs individuals’ thinking and behavior, have garnered researchers’ significant interest due to their close relationship with concurrent and long-term academic outcomes (e.g., Fuhs et al., 2014; Schmitt et al., 2017). EF is an umbrella term encompassing various cognitive skills involving planning, monitoring, and controlling cognitive functions to achieve goal-oriented tasks (Diamond, 2013). While the conceptualization of EF may vary across studies, there is widespread agreement that EF consists of three components (Miyake & Friedman, 2012): (1) inhibition, referring to the ability to intentionally withhold impulsive responses and prioritize relevant information for task processing; (2) attention shifting, involving the flexible transition between multiple tasks, operations, or mental sets; and (3) working memory, which entails the maintenance, updating, and manipulation of information in the mind. Baddeley and Hitch (1974) further specified working memory into two separate components: verbal working memory, primarily concerning verbalizable information, and visual working memory, primarily concerning visuospatial information. Inhibition, attention shifting, and working memory are suggested to be distinct but interconnected (e.g., Brocki & Tillman, 2014; Diamond, 2013; Miyake et al., 2000; St. Clair-Thompson & Gathercole, 2006; Zuber et al., 2019). In Miyake et al. (2000)’s classic study of EF, the components of EF shared some common variance, implying that they were correlated. Therefore, EF has been widely conceptualized as a latent variable (e.g., Brydges et al., 2014; Karr et al., 2018; Spencer et al., 2020).

In recent years, an increasing number of studies have reported a strong association between EF and word recognition, not only in alphabetic languages (e.g., English: Burgoyne et al., 2019; Cartwright et al., 2019; French: Colé et al., 2014; Greek: Altani et al., 2017; Protopapas et al., 2007; Dutch: Van der Sluis et al., 2007) but also in languages such as Chinese (e.g., Chung & McBride-Chang, 2011; Chung et al., 2018; Ren et al., 2022; Yang & Qiao, 2021). Experimental evidence suggests that EF plays a critical role in word recognition processing. In an fMRI study by Aboud et al. (2016), researchers found that, in addition to the activation of areas related to domain-specific skills such as orthographic and semantic processing, an area related to EF was also activated during word recognition. Furthermore, Zhou and McBride (2018) conducted a study among students with word recognition deficits and reading comprehension deficits from Grades 3 to 5. They found that children with word recognition deficits exhibited poorer performance in both working memory and inhibition, whereas children with reading comprehension deficits only showed lower competence in working memory. Indeed, EF has been theorized to function holistically in supporting word recognition in various ways, such as containing the visual form of a word in working memory for mapping it with the sound and meaning, suppressing the activation of irrelevant information during word recognition through inhibition skills, and continuously switching between form-sound correspondence rules under the control of attention shifting (e.g., Chung & McBride, 2011; Pan & Lin, 2023).

EF has also been found to play an even more important role in L2 word recognition (e.g., Bialystok et al., 2003; Gastmann & Poarch, 2022; Grant et al., 2015; Poarch & Van Hell, 2012). In the fMRI study by Grant et al. (2015), L2 learners showed greater engagement of the control system (related to EF) while reading, compared to their native peers. Successful L2 word recognition requires EF to manage interference from L1. Gastmann and Poarch (2022) reported that inhibition was particularly important for recognizing noncognate L2 words among German–English bilingual kindergarteners aged 4 to 6, which was probably because its assistance in inhibiting interference from L1 noncognate word forms. According to Kroll and Stewart (1994), the representations of L2 words are accessed via the L1 translation equivalent due to weaker L2-to-concept links in beginning L2 learners. However, the activated L1 representation may impede identification of noncognate words due to the lack of form overlap. Inhibition could therefore demonstrate particular importance to L2 word recognition. Additionally, it was argued that EF skills (e.g., working memory and attention shifting) could aid in the fast-mapping process, where new words are quickly learned and stored (Perikova et al., 2024). In other words, the importance of EF in L2 word recognition may also stem from its role in the acquisition of L2 words.

Despite the previously identified critical importance of EF, the underlying mechanism linking EF and word recognition has not been fully elucidated. Considering the complexity of Chinese orthography and its word system, the interaction between EF and domain-specific skills (i.e., morphological awareness and orthographic awareness) in word recognition should be examined and discussed among Chinese learners. Moreover, previous studies have suggested differences in word acquisition (e.g., Hamada & Koda, 2011; Jiang, 2018) and word reading processes (e.g., Davis & Bistodeau, 1993; Zhang, 2017) between native and L2 learners. To the best of our knowledge, the role of EF in word recognition has not been compared in parallel between native and L2 learners, particularly in Chinese.

### Importance of Linguistic Skills to Word Recognition

According to classic theories of word recognition (e.g., Dual-Route Cascaded (DRC) Model, Coltheart et al., 2001; Lexical Constituency Model, Perfetti et al., 2005), orthographic awareness and morphological awareness have been identified as two essential linguistic skills that underpin the process of Chinese word recognition (Chen & Zhao, 2022; Liu et al., 2017; Yeung et al., 2011; Yeung et al., 2013). Orthographic awareness in Chinese refers to understanding radical functions (i.e., the semantic/phonological clues provided by radicals) and the orthographic conventions of Chinese characters (Ho et al., 2003; Leong et al., 2011). It has been theorized to facilitate the complex form-sound-meaning correspondence in Chinese, infer the meaning/sound of unfamiliar characters (e.g., Chen, 2021; Li et al., 2012), and be involved in learning new characters (e.g., Tong et al., 2019). Empirical evidence has supported that orthographic awareness is a significant contributor to Chinese word recognition among both native learners (e.g., Li et al., 2012; Fung & Chung, 2020; Tong et al., 2019) and CSL learners (e.g., Chan et al., 2021; Jiang & Wu, 2022). Morphological awareness is the ability to manipulate morphemes (i.e., the most basic meaningful word constituents) (McBride-Chang et al., 2003; Tong et al., 2017). Morphological awareness primarily supports word recognition by allowing learners to infer the meaning of an unfamiliar word by integrating the knowledge of morphemes from learned words (Choi et al., 2018; Liu et al., 2013). Moreover, it has been found to assist learners in distinguishing homophones (Shu et al., 2006). Due to the large number of homophonic morphemes and compound words in Chinese, morphological awareness is of great importance to Chinese word recognition among both native learners (e.g., Chung & McBride-Chang, 2011; Pan & Lin, 2023) and CSL learners (e.g., Choi et al., 2018; Koda, 2021).

Nevertheless, it is noticeable that the effect of orthographic awareness and morphological awareness on Chinese word recognition may differ between native and CSL learners. L2 learners were found to be more easily interfered with the written form of characters compared to their native counterparts (e.g., Jiang & Wu, 2022). Specifically, L2 learners demonstrated confusion when encountering words that were orthographically similar but not semantically related, such as the word pair *freeze* and *free*. Additionally, in the study by Zhang (2017), which explored the contribution of morphological awareness to word recognition among both native and CSL children (N = 180 and 115 respectively), the importance of morphological awareness among CSL children was not as prominent as it was among native Chinese learners. Due to less exposure to spoken and written Chinese, CSL learners may not develop domain-specific skills to the same level as their native Chinese counterparts. As argued by Zhang (2017), rather than relying less on morphological awareness, CSL learners may face challenges in employing morphological awareness in the process of word recognition due to the disparate learning experiences of Chinese. Nevertheless, the relative importances of domain-specific skills in word recognition between CSL learners and native counterparts are awaiting for more thorough examination.

### Relationship between EF, Domain-specific Skills, and Word Recognition

Previous findings have suggested hierarchical associations between EF, domain-specific skills (i.e., orthographic awareness and morphological awareness), and word recognition. Specifically, EF may serve as an enhancer to support the employment of domain-specific skills during word recognition (e.g., Kim, 2017). Generally, EF is critical in employing orthographic awareness in Chinese word recognition (e.g., Hamilton et al., 2016; Huo et al., 2021; Liu et al., 2019; Pan & Lin, 2023) for the following reasons. First, activating and integrating orthographic information necessitates attention shifting to efficiently switch between orthographic rules and coordinate multiple pieces of orthographic information (Fong & Chung, 2020; Pan & Lin, 2023). Second, enhanced working memory can hold extracted visual features and activated orthographic knowledge in mind, which is critical for strategically associating the form and sound of characters/words (Hamilton et al., 2016). Furthermore, inhibition may be necessary given that the clue provided by a radical can often be unreliable. Due to the complexity of Chinese orthography, such as the less correspondence between written form and sound/meaning, the role of EF skills could be more prominent in Chinese. Indeed, in a study conducted among 6-year-old kindergarteners by Fong and Chung (2020), attention shifting was found to support word recognition by effectively manipulating orthographic knowledge between characters (*β* = 0.08; *p* < 0.01). Additionally, Huo et al. (2021) found that orthographic awareness mediated the relationship between working memory and word recognition among an average of 197 children aged 5 (*β* = 0.11; *p* < 0.01).

Regarding the interaction between morphological awareness and EF, a few findings have also suggested an interaction between EF and morphological awareness in word recognition (e.g., Colé et al., 2014; Huo et al., 2021; Pan & Lin, 2023). For example, Pan and Lin (2023) found that EF skills significantly contribute to word reading via its function on morphological awareness among native Chinese learners. As explained by Pan and Lin (2023), EF skills are important given that readers need the working memory to process morpheme meanings and integrate them into word meanings. Moreover, as aforementioned, the number of homonyms among single-character words in Chinese is large. Meanwhile, many compound words embed the same morpheme. Previous studies have suggested that homophonic morphemes or words embedding the same morpheme are tentatively activated during the word recognition process (e.g., Ferrand & Grainger, 2003; Jiang et al., 2020; Newman, 2012). Accordingly, we may assume that EF is necessary for utilizing the most appropriate morphological knowledge to recognize characters and words in Chinese.

## The Present Study

The present study aims to explore the underpinning role of EF in word recognition among both native Chinese learners and CSL learners. Specifically, how EF supports the manipulation of two essential linguistic skills of word recognition, namely orthographic knowledge and morphological awareness, will be explored and discussed. The involved CSL participants in the present study are mostly the descendants of Southeast Asian immigrants with languages such as Urdu, Hindi, or Tagalog as their first or family language. The lingua franca for social communication among them has predominantly been English (Wang & Tsung, 2022; Zhang et al., 2010). Furthermore, many of them reside in culturally segregated communities and communicate with their families in their heritage language (Loh et al., 2018). Due to limited exposure and input, these young CSL learners struggle with developing Chinese reading skills such as orthographic awareness and morphological awareness, and achieve significantly lower levels of Chinese language proficiency (Ku et al., 2005; Wong, 2018; Wong & Shiu, 2009; Zhou & McBride-Chang, 2018). In terms of school learning, there are issues of lacking appropriate teaching materials and tailor-made teaching approaches. For example, the “observe and memorize” approach (i.e., asking learners to observe and rote-learn character shapes) is widely employed to teach Chinese characters to CSL learners (Loh et al., 2015; Chang, 2009). Although this approach could be effective among native learners, Loh et al. (2017) reported that it could reduce CSL learners’ motivation, making it less effective. Moreover, a significant number of CSL learners in Hong Kong study in “mainstream schools” intended for native children (Wang, 2021). CSL learners are placed in the same classes as their native peers despite having a limited level of Chinese compared to their native counterparts, which causes higher levels of anxiety and frustration (Gao & Shum, 2010). Investigation into the difference between Chinese and CSL students’ characteristics in word recognition would deepen teachers’ understanding of learning needs from different learning groups, allowsing teachers instructing appropriate teaching contents and implimenting tailor-made approaches.

To sum up, two research questions are proposed in the present study:

(1) Will EF skills indirectly affect Chinese word recognition through orthographic awareness and morphological awareness apart from the direct effect?

(2) Do the direct and indirect effects of EF on word recognition vary between native Chinese leaners and CSL learners?

**Fig. 1:**
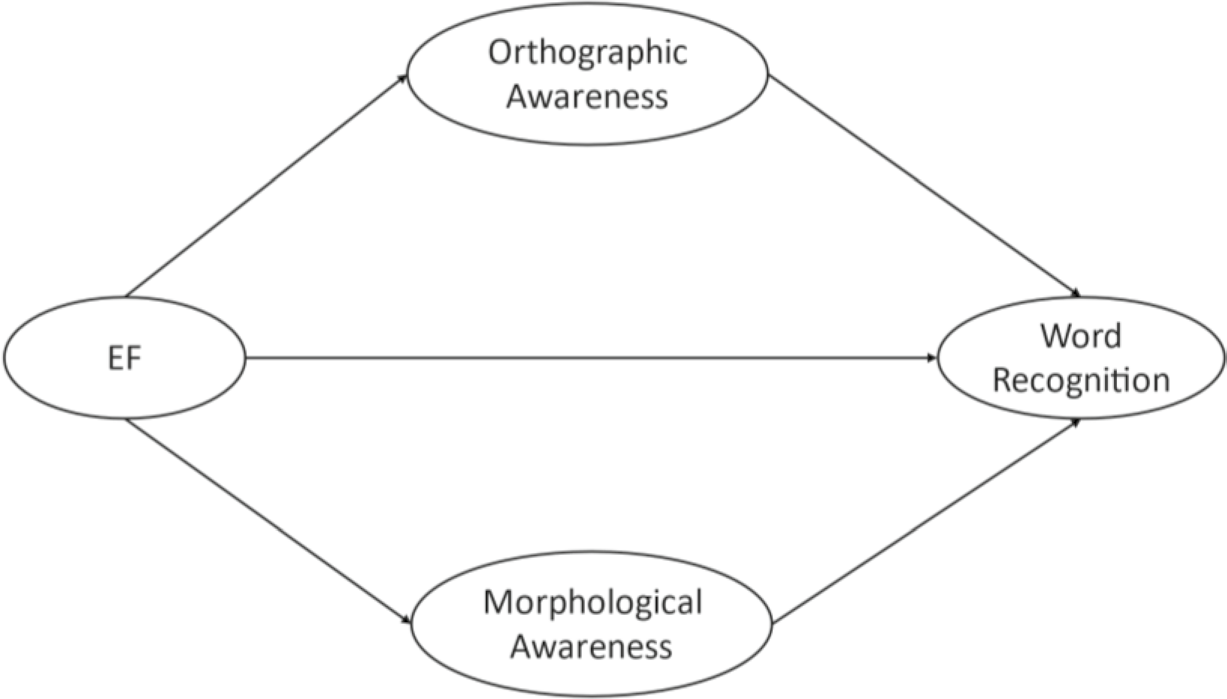
Theoretical model of the indirect effects of EF on word recognition via orthographic awareness and morphological awareness

The hypothesized model of the present study is demonstrated in Fig. 1, with all variables constructed as latent variables.

## Research Methods

### Participants

Considering the lagged developed domain-specific skills of CSL learners, the participants of our study were all from Grade 4, who have been considered to develop a certain level of domain-specific skills (e.g., Derwing & Munro, 2013; van Koert et al., 2023). 419 Chinese and 204 CSL Grade 4 students (mean age = 9.93 years, SD = 1.09) from 6 local Hong Kong primary schools involved in the present study. The schools were all subsidized schools under the same central curriculum framework launched by the Education Bureau of Hong Kong. Among them, 298 were boys, and 325 were girls. Regarding CSL learners, around 91% of the students have been to Hong Kong for more than 7 years. Reported by their schoolteachers, their mother/heritage languages are the languages they use to communicate with family members in daily life. Given that all the participants were from the same grade, and no special education needs were reported, the present study did not control age and non-verbal intelligence in the following analysis.

### Instruments

The participants were invited to complete a battery of tests, measuring word recognition, EF (including inhibition, attention shifting, and working memory), orthographic awareness, and morphological awareness. All tasks were conducted by well-trained research assistants. Five-minute breaks were given between every two tasks, and the whole test lasted approximately 1.5 hours in total.

#### Word Recognition

Both single-character reading and two-character word recognition were evaluated in the word recognition task due to increasing evidence suggesting the two processes can be different (e.g., Pan et al., 2021; Wang & McBride, 2016). The task followed the design of Wang and McBride-Chang (2016). A total of 150 items were included, with 50 single characters and 100 two-character words. The order of characters/words was arranged according to their frequency, with an overall difficulty level progressing from easy to hard. During the test, the participants were asked to read aloud the printed characters/words, starting with higher frequency items (easier) and moving to lower frequency items (harder). Fifty percent of the tested characters and words were learned items, sampled from Chinese textbooks for primary school students, while the other half were unlearned items selected from Chinese textbooks for secondary school students (Grade 7 to Grade 9). One point was given if the pronunciation of the item (i.e., single-character/two-character word) was entirely correct. Half points were given if the tone/rhyme of a single character was inaccurate, or if one of the characters was incorrectly pronounced (i.e., the consonant, rhyme, and tone were all incorrected) in a two-character word. The maximum score was 150. The internal consistency (Cronbach’s alpha) of the task was 0.99.

#### Executive Functions

*Inhibition.* The online Color-Word Stroop Task adapted from Van Der Elst et al. (2008) was used to measure the ability of inhibition. Three subtasks were included, word task (Stroop I), color task (Stroop II), and color-word task (Stroop III). In the word task, the participants were asked to respond to the color of Chinese character shown on the screen by pressing buttons on the keyboard (“A- 紅red”, “G-藍blue” or “L-黃yellow”). In the color task, participants were asked to choose the correct color of the patch “XXXX” shown on screen from three options. In the color-word task, the stimuli were Chinese words “紅”, “藍” and “黃” that were randomly printed with a different color other than the meaning of word. There were 51 formal trials in every subtask, and 3 practice trials were given before the formal task. Participants were required to make a response to each trial within 2500 ms, and their reaction time was recorded. The final result of this task was calculated by the formula: Interference time = Stroop III—[(Stroop I + Stroop II) / 2]). The Cronbach’s alpha of the task was 0.86.

*Attention Shifting.* Wisconsin Card Sorting Test adapted from Grant and Berg (1948) was used for measuring attention shifting. During the test, there were four key cards varying from color to shape presented at the top of the screen, including a red circle, two green triangles, three yellow crosses, and four blue stars. The response cards were presented one at a time at the left bottom of the screen. The participants were asked to match the response cards with any of the four key cards. The cards could be matched by color (red, yellow, and blue), shape (stars, crosses, triangles, and circles), and number (one to four identical figures). Each sorting rule represented one category. Without being told the sorting rules, the participants could only infer the rules from the feedback (whether the matching was right or wrong) received after each time they made a choice. Two trials were given before the official test. The sorting rules were altered every 10 items. The total number of items was 60. The completed categories were recorded as indices for further analysis. The Cronbach’s alpha of the task was 0.66.

*Working Memory.* Following the theory of Baddeley and Hitch (1974), both *verbal working memory* and *visual working memory* were measured. The backward digit span task adapted from WISC-IV (Wechsler, 2003) was used to evaluate *verbal working memory*, in which 14 trials and 2 practice trials were included. In each trial, a sequence of digits was audibly given at a rate of one digit per second, and students were required to listen carefully to the digits and recall them in reverse order afterward. For example, when hearing “3–5-1”, the participants should click “1–5-3” on the nine-box grid shown on the screen. The response must be given within 30 seconds after hearing the digits. The sequence of the digits was randomly generated. The span size started from 2 digits and went up to a maximum of 8. Each span size had two trials. If students successfully complete 2 successive trials of the same length, the number of digits for the subsequent trial would increase by one. The task would end if participants failed on two successive trials with the same length of digits. The ability of verbal working memory was represented by multiplying the longest span with the number of correct trials. The Cronbach’s alpha of the task was 0.84.

The task for assessing *visual working memory* was adapted from Van de Weijer-Bergsma et al. (2015). During the test, a nine-box grid was presented in the center of the screen. In each trial, the pattern of a lion appeared one at a time in several positions on the nine-box grid, and the subjects were asked to recall the showed-up sequence in a reversed order by clicking the box in the grid. The number of lions increased from two to eight sequentially, and each length contained two items. The response must be given within 30 seconds after hearing the digits. The tasks would be terminated if two items in the same difficulty level were wrongly answered. The result of this task was calculated by multiplying the longest recalled span with the number of correct answers. Two practice trials were given before the real test. The score was the product of the longest span and number of correct trials. The Cronbach’s alpha of the task was 0.85.

#### Orthographic Awareness

The two orthographic tasks were adopted from Leong et al. (2011), which were designed to measure Chinese orthographic awareness among both native and non-native Chinese learners. Given that our study also focuses on both native and CSL learners, these two types of orthographic tasks were used.

*Orthographic Choice Task.* This task aimed to measure learners’ sub-lexical orthographic awareness, which asked students to choose the correct written form of words. A total of 20 items were included in the task. 10 items paired with characters that were consistent with their phonological/semantic radicals, for example: 青山 [cing1 saan1, green mountain]—蜻山 [cing1 saan1, homophonic pseudoword]. The other 10 items were paired with characters that are not consistent with their phonological/semantic radicals, for example, 米飯[mai5 faan6, rice] – 米反 [mai5 faan2]. All real words and the characters in pseudowords were randomly selected from the Lexical Items for Fundamental Chinese Learning in Hong Kong Schools (Hong Kong Education Bureau, 2003) (https://www.edbchinese.hk/lexlist_ch/). One point was given to each correct answer. The Cronbach’s alpha of the task was 0.96.

*Orthographic Choice in Context.* The tasked aimed to evaluate students’ lexical orthographic knowledge by testing their knowledge of word forms in a situation of reading. The task consisted of 18 short sentences with blanks. Students should first determine the missing word in the blank with the context provided by the sentence, and then choose the correct orthographic form of word among four options. A sample was provided as follows: 早上____照進課室 (*____sheds into the classroom in the morning*). Four options were provided: (1) 洋光 (*ocean-light*, pseudoword); (2) 陽光(*sunshine*) (3) 揚光 (*blowing-light*, pseudoword) (4)羊光 (*sheep-light*, pseudoword). In each item, all options were two-character words, and three of them were distractors that are pseudowords that were visually and phonologically resembled to the correct answer. All tested words were randomly selected from the Lexical Items for Fundamental Chinese Learning in Hong Kong Schools (Hong Kong Education Bureau, 2003) (https://www.edbchinese.hk/lexlist_ch/). 18 items were included in this task, with one point per item. The Cronbach’s alpha of the task was 0.95.

#### Morphological Awareness

*Morpheme Discrimination Task.* This task was adapted from Ku and Anderson (2003) was used in the present study. In each item, there were three words that all contained the same morpheme. The common parts in two words had the same/similar meaning, while there was an odd word whose shared part was semantically different from the other two words. The task for the participants was to select the odd word. For example, among the words 草地 *grass*, 草原 *grassland*, and 草稿 *draft version*, the odd word went to 草稿 *draft version*, because 草 *draft* in 草稿 *draft version* did not equal to the meaning of 草 *grass* in the first two words. All involved words in this task were randomly selected from the Lexical Items for Fundamental Chinese Learning in Hong Kong Schools (Hong Kong Education Bureau, 2003) (https://www.edbchinese.hk/lexlist_ch/) to ensure they were all familiar to the participants. This task had thirty items, with one point for each item. The total score was 30. The Cronbach’s alpha of the task was 0.95.

*Morpheme Recognition Task.* This task was also adapted from Ku and Anderson (2003). During the task, students were asked to judge whether the first character of a word was semantically related to the whole word’s meaning. For example, when being presented with the word 書架 *bookshelf*, a “yes” answer should be given because the first component character 書 *book* was related to the meaning of the 書架 *bookshelf*. All involved words in this task were randomly selected from the Lexical Items for Fundamental Chinese Learning in Hong Kong Schools (Hong Kong Education Bureau, 2003) (https://www.edbchinese.hk/lexlist_ch/), which were all familiar to the participants. Thirty items were included, and one point was given to each correct answer. The Cronbach’s alpha of the task was 0.90.

### Data Analysis Strategies

Students’ performance in all tasks was calculated and entered SPSS 28 for further statistical analysis. Descriptive analysis was first performed to check the overall characteristics of the dataset, in which normalities of variables were checked. Additionally, a t-test was conducted to compare students’ performances on the measured tasks between the native and CSL groups. Correlation analysis was further conducted to obtain the rough links among variables. Later, structural equation modelling (SEM) was used to determine the direct and indirect effects of EF on word recognition by AMOS 29. Once the basic model was established, we followed the procedure of multi-group comparison (Kline, 2005), and tested three levels of measurement invariance with increasing constraints. They are configural invariance model (latent variables contain same factor structure across groups), metric invariance model (latent variables have equal factorial loading for each item), and scalar invariance model (both the factor loadings and intercepts of items are assumed equal). Given that full scalar invariance is difficult to achieve in many cases (Putnick & Bornstein, 2016), partial scalar model could be used for the subsequent structural invariance tests. To evaluate the model fit, we took multiple criteria into account, including CFI ≥ 0.90 and SRMR ≤ 0.08 (Hu & Bentler, 1999). RMSEA is usually an important indicator of model fit, and we referred to Fabrigar et al. (1999)’s suggestion (i.e., values less than 0.05 constitute good fit, values in the 0.05 to 0.08 range acceptable fit, values in the 0.08 to 0.10 range marginal fit, and values greater than 0.10 poor fit). It is important to note that while one criterion alone may not be entirely satisfactory, we consider multiple criteria to assess the overall acceptance of the model. In comparing various models, in addition to the traditional way by checking the change of is χ2, we took changes in CFI of 0.02 and RMSEA of 0.03 as criteria to determine if the fit index changed significantly (Klime, 2015; Putnick & Bornstein, 2016).

To determine the significance of the indirect effects in each model, we performed the bias-corrected bootstrapping by 2000 random samplings with replacements at 95% of confidence level. The indirect effects were assumed to be significant when zero was beyond the confidence interval (CI).

## Results

### Descriptive Data

Students’ performance in all tasks is shown in Table 1. The variables’ normality for the two groups of students was checked by examining values of skewness and kurtosis according to the criteria set by Kline (2015) (i.e., less than 3 for skewness and 10 for kurtosis). While all variables were considered normally distributed, it was found that the single character reading has higher values of skewness and kurtosis (-3.76 and 15.57 respectively). Given that Tabachnick and Fidell (2001) argued that the non-normality issue could be less severe in a large sample size (more than 100), therefore, we followed previous research practices on word recognition (Choi et al., 2018) and kept the raw data of single character reading and completed the other analysis.

**Table 1:** Descriptive statistics

	Mean (Native)	SD (Native)	Mean (CSL)	SD (CSL)	*t*	*df*	*p*
SCR	43.13	3.94	22.20	17.08	− 17.28	213.59	^***^
TWR	91.77	16.03	34.50	37.77	− 20.77	239.29	^***^
CR	134.90	18.93	56.70	53.94	− 20.11	227.67	^***^
OC	18.68	3.37	12.87	6.90	− 11.39	251.67	^***^
OCC	17.21	3.65	9.49	6.79	− 15.21	261.75	^***^
MD	22.43	6.75	12.70	9.32	− 13.31	310.26	^***^
MR	20.26	5.87	14.31	7.98	− 9.46	313.48	^***^
Inhibition	0.10	0.07	0.11	0.07	1.84	621.00	−
AS	1.88	0.76	1.77	0.75	− 1.80	328.77	−
WM (verbal)	33.93	18.13	30.65	15.69	− 2.35	369.93	^**^
WM (visual)	38.38	17.09	33.06	15.60	− 3.91	351.34	^***^

SCR, single character reading; TWR, two-character word recognition; CR, character reading (total); OC, orthographic choice; OCC, orthographic choice in context; MD, morphological discrimination; MR, morphological recognition; AS, attention shifting; WM (verbal), verbal working memory; WM (visual), visual working memory

*p < .05, ** *p* < .01, *** *p* < .001

### Correlation Analysis

Pearson’s correlations among all variables in native and CSL learners are demonstrated in Table 2.

**Table 2:** Correlations between variables among native and CSL students (N = 419 and 204 respectively)

CSL	SCR	TCR	CR	OC	OCC	MD	MR	Inhibition	AS	WM (verbal)	WM (visual)
Native											
SCR	1	.92^**^	.96^**^	.68^*^	.82^**^	.75^*^	.56^**^	− .20^**^	.11	.37^**^	.30^**^
TCR	.68^**^	1	.99^**^	.61^**^	.86^**^	.81^**^	.55^**^	− .27^**^	.15^*^	.42^**^	.36^**^
CR	.78^**^	.99^**^	1	.64^**^	.86^**^	.80^**^	.56^**^	− .25^**^	.13^*^	.41^**^	.34^**^
OC	.21^*^	.28^**^	.28^**^	1	.78^**^	.72^**^	.75^**^	.01	− .02	.20^**^	.08
OCC	.43^**^	.51^**^	.52^**^	.78^**^	1	.90^**^	.73^**^	− .12	.11	.35^**^	.24^**^
MD	.41^**^	.49^**^	.50^**^	.57^**^	.74^**^	1	.80^**^	− .13^+^	.11	.36^**^	.26^**^
MR	.30^**^	.38^**^	.38^**^	.53^**^	.66^**^	.69^**^	1	.07	− .02	.19^**^	.05
Inhibition	− .15^**^	− .17^**^	− .18^**^	.01	− .03	− .10^*^	− .03	1	− .20^**^	− .16^*^	− .23^**^
AS	.24^**^	.20^**^	.22^**^	.078	.15^**^	.25^**^	.18^**^	− .11^*^	1	.26^**^	.32^**^
WM (verbal)	.29^**^	.28^**^	.29^**^	.13^**^	.25^**^	.30^**^	.24^**^	− .10^*^	.21^**^	1	.38^**^
WM (visual)	.22^**^	.22^**^	.23^**^	.08	.16^**^	.18^**^	.10^*^	− .13^**^	.24^**^	.20^**^	1

The data for native students is displayed in the bottom-left of the table, while the data for CSL learners is displayed in the top-right of the table

SCR, single character reading; TWR, two-character word recognition; CR, character reading (total); OC, orthographic choice; OCC, orthographic choice in context; MD, morphological discrimination; MR, morphological recognition; AS, attention shifting; WM (verbal), verbal working memory; WM (visual), visual working memory

**correlation is significant at the .01 level (2-tailed); ^*^ correlation is significant at the .05 level (2-tailed). ^+^correlation is marginally significant (*p* < .06)

Among native Chinese learners, all independent variables were significantly correlated with word recognition. The coefficient values between word recognition and two orthographic awareness (i.e., sub-lexical orthographic awareness and lexical-orthographic awareness) were 0.28 and 0.52 respectively, *p* < 0.01. Moderate correlations were found between two morphological awareness tasks (morpheme discrimination and morpheme recognition) and word recognition, with *r* equal to 0.50 and 0.38 respectively, *p* < 0.01. Correlations between word recognition and EF skills were relatively weak. A negative correlation was found between word recognition and inhibition, *r* = -0.18 (*p* < 0.01). Because the lower value of inhibition represented better skill, such a value indicated a positive relationship between inhibition and word recognition. For other EF skills’ correlations with word recognition, all coefficients were significant: attention shifting, *r* = 0.22, verbal working memory (*r* = 0.29), and visual working memory (*r* = 0.23). All *p* values < 0.01.

Among CSL learners, all variables were found to relate significantly to word recognition as well, whereas the correlations were much stronger than what were found among native Chinese learners. Strong correlations were found between word recognition and orthographic awareness, with *r* equal to 0.64 (sub-lexical orthographic awareness) and 0.86 (lexical orthographic awareness) respectively (*p* < 0.01). Moderate to strong correlations were found between word recognition and morphological tasks. The score of two morphological awareness tasks, i.e., morpheme discrimination and morpheme recognition tasks also strongly correlated with word recognition, *r* = 0.80 and 0.56 respectively (*p* < 0.01). Regarding EF skills, weak correlations were found with word recognition in general. The correlation between word recognition and inhibition, attention shifting, and visual working memory were -0.25 (*p* < 0.01), 0.14 (*p* < 0.05), and. 34 (*p* < 0.01) respectively. In addition, verbal working memory was found to be moderately correlated with word recognition, *r* = 0.41 (*p* < 0.01).

### Direct and Indirect Effect of EF on Word Recognition

**Fig. 2:**
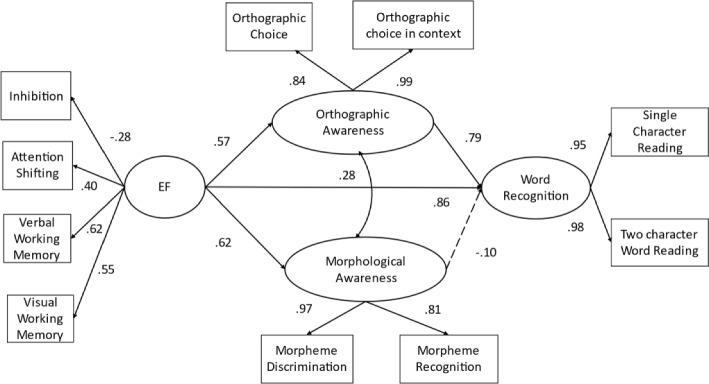
Direct and indirect effect of EF on Chinese word recognition. *Note*. Solid lines indicate significant paths, while dashed line represents non-significant path

To better determine the direct and indirect effect of EF on word recognition, we used four latent variables to construct the path model. The latent variables included EF (4 indicators), orthographic awareness (2 indicators), morphological awareness (2 indicators), and word recognition (2 indicators). The path model of EF’s direct and indirect effect on word recognition is presented in Fig. 1. The model fit well as the measurement model, χ2(36) = 179.98, RMSEA = 0.08, CFI = 0.97, SRMR = 0.04. Based on the model, EF could have a direct effect on word recognition, *β* = 0.28. Meanwhile, it could also predict the orthographic awareness (*β* = 0.57), which further impacted word recognition (*β* = 0.79). The indirect effect was 0.45(0.57 * 0.79), which was significant at 0.01 level, 95% CI [0.35, 0.60] (Fig. 2).

### Effect of EF on Word Recognition between CSL and Chinese Native Students

Before performing the group invariance analysis, we first performed the model in separate groups of students. For the CSL group, the model presented acceptable values in most fit indices, χ2(36) = 141.13, RMSEA = 0.91, CFI = 0.93, SRMR = 0.06. For the native-student group, χ2(36) = 52.51, RMSEA = 0.04, CFI = 0.99, SRMR = 0.04. Therefore, the model was applicable to two groups of students.

Next, we conducted the multi-group comparison following the procedure recommended by Kline (2015). The unconstrained (configural) model had a good fit, χ2(72) = 193.83, RMSEA = 0.06, CFI = 0.96, SRMR = 0.07. The subsequent measurement weight (metric) model also fit well, χ2(66) = 264.71, RMSEA = 0.07, CFI = 0.94, SRMR = 0.08, suggesting that the model was comparable across two groups. However, the fit index for the subsequent measurement intercepts (scalar) model became worse substantially, χ2(56) = 527.81, RMSEA = 0.010, CFI = 0.86, SRMR = 0.20, which suggested that intercepts for some variables were variant across groups. According to Putnick and Bornstein (2016), a partial scalar model was allowed if at least half variables’ intercepts were invariant. After checking each variable’s intercept, we decided to allow half (i.e., two orthographic variables, one morphological awareness, and two-word recognition variables) variables’ intercepts unconstrained due to the distinct performance across two groups. Therefore, the partial-scalar model had an acceptable fit index, χ2(61) = 394.06, RMSEA = 0.09, CFI = 0.90, SRMR = 0.13.

**Fig. 3:**
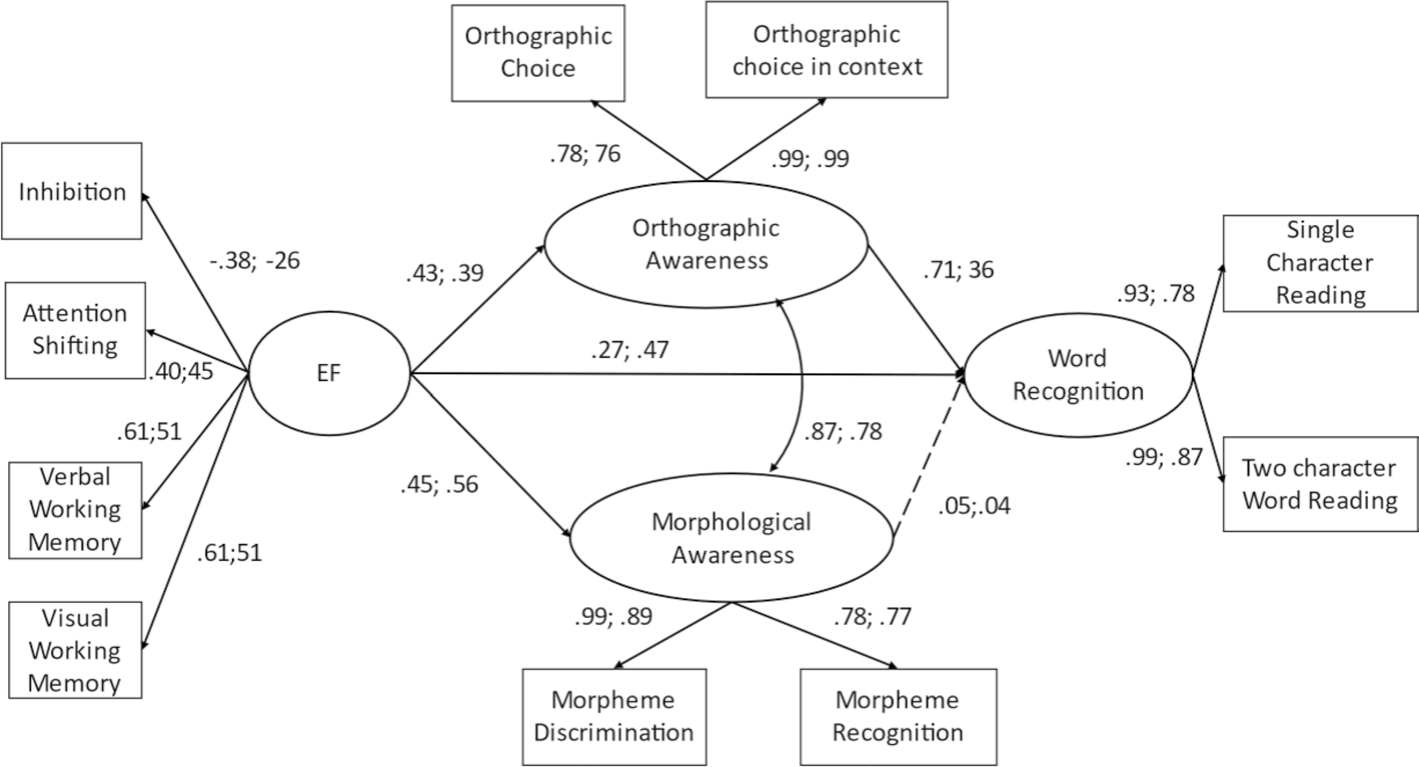
Group comparison of the direct and indirect effect of EF on chinese word recognition between native and CSL learners. *Note*. Solid lines indicate significant paths, while dashed line represents non-significant path. Numbers on the left side of each path correspond to the results for CSL learners, and numbers on the right side represent the results for native learners

Based on the partial-scalar model, we performed structural weights invariance analysis. Given that the structural invariance model did not fit well, χ2(56) = 515.80, RMSEA = 0.10, CFI = 0.86, SRMR = 0.17, we inferred that some paths could be varied across groups. Therefore, we performed more analysis by constraining one path each time and found that two paths could significantly change the χ2 of the model. They were *EF → orthographic awareness* (∆χ2 = 10.59, *p* < 0.01), and *orthographic awareness → word recognition* (∆χ2 = 15.94, *p* < 0.01). In other words, the coefficients of these two paths in CSL students were significantly higher than Chinese native students. For the indirect effect of EF on word recognition through orthographic awareness, it was found that such indirect effect was 0.31 among CSL group, 95%CI [0.18, 0.50], *p* < 0.01 and 0.14 among Chinese native students, 95%CI [0.01, 0.29], *p* < 0.05 (Fig. 3).

## Discussion

This study examined how EF contributes to Chinese word recognition through domain-specific skills (i.e., orthographic awareness and morphological awareness) among both native and CSL primary students. Two major findings emerged from the analysis: (1) apart from direct effect, EF could also indirectly impact word recognition via orthographic awareness; while the indirect path via morphological awareness was not significant; (2) both direct and indirect paths from EF to word recognition were applicable in both groups, whereas the indirect effect of EF via orthographic awareness was found to be stronger in CSL learners. The present study is perhaps the first study comparing the effect of EF on word recognition between both native and CSL learners, which not only enriches our insight into word recognition among different learning groups but also provides pedagogical inspiration.

### Direct and Indirect Effect of EF on Word Recognition

By conceptualizing EF as a latent variable, we were able to determine the contribution of EF as an integrated mechanism of domain-general skills in word recognition. We found both direct and indirect contributions of EF to word recognition in the context of Chinese. Previous studies that used reading comprehension as the outcome variable reported that the direct effect of EF would be fully mediated by domain-specific skills, including orthographic awareness and morphological awareness (e.g., Pan & Lin, 2023; Yang et al., 2019). However, the present study found that the direct effect of EF on word recognition remained significant, even when considering the mediation effect of domain-specific skills. This finding aligns with the study by Zhou and McBride (2018), which found that EF was more involved in word recognition than in reading comprehension. As a fundamental cognitive skill, EF may not have a direct impact on reading comprehension, but it may function more significantly on lower-level literacy skills such as word recognition (Kim, 2017). We may therefore observe the significant direct effect of EF on word recognition. Additionally, our results may be related to the characteristics of Chinese words. For example, previous studies have reported that students tend to transpose the two characters within a compound word (e.g., 蜜蜂 *bee* and 蜂蜜 *honey*) when reading Chinese words (e.g., Gu et al., 2022; Sun et al., 2021; Xu et al., 2014). Unlike alphabetic orthography, Chinese does not have prominent spaces between words. Therefore, as proposed by Sun (2021), word recognition in Chinese may require more EF skills to monitor the reading process and ensure the correct correspondence between the written input and the sound/meaning of the word, which lead to the remarkable direct effect of EF.

Regarding the indirect effect, EF is found to contribute to word recognition through its impact on orthographic awareness. While the importance of orthographic awareness in word recognition is undeniable (e.g., Chan et al., 2021; Liu et al., 2022; Tong et al., 2019), our study further explains how such an effect takes place. Due to the complex orthography of Chinese, previous studies suggested that the word recognition could be effortful. For instance, some studies reported that radical knowledge could assist readers in recognizing Chinese characters more quickly (e.g., Ding et al., 2004; Taft, 2006; Zhang et al., 2020), however, other studies reported distraction effects (i.e., longer reaction times when the priming and target characters share the same radical) in recognizing Chinese characters (e.g., Chen & Yeh, 2015; Li et al., 2017; Liu et al., 2023). The significant indirect effect of EF on word recognition via orthographic awareness implies that learners with better EF would be able to utilize the most relevant and appropriate orthographic knowledge to facilitate word recognition, while learners with weaker EF might probably be more influenced by the distraction effects caused by complex orthographic system as mentioned earlier.

However, morphological awareness did not mediate the association between EF and word recognition for both native and CSL learners. Despite significant correlations found between morphological awareness and word recognition in our study, morphological awareness did not predict word recognition in the SEM model. One possible explanation could be that Chinese learners primarily rely on visual skills during the early stages of learning (Hulme et al., 2017). Yang et al. (2019) discovered that among various linguistic skills, orthographic awareness exhibited the strongest influence on Chinese word recognition (*β* = 0.24), while the influence of morphological awareness was the weakest (*r* = 0.04). Additionally, the insignificant effect of morphological awareness on word recognition might also be related to the complex relationship between morphological awareness and orthographic awareness in Chinese. For instance, Liu et al. (2017) reported that orthographic awareness could mediate the association between morphological awareness and word recognition. In the present study, both sub-lexical and lexical orthographic awareness were measured. Therefore, the variance explained by morphological awareness in word recognition may be overshadowed by the powerful effect of orthographic awareness on word recognition among Chinese learners.

### Difference in Effect of EF on Word Recognition between Chinese Native and CSL Students

Although the role of domain-specific skills in word recognition has been extensively discussed among CSL learners (e.g., Choi et al., 2018; Koda, 2021; Zhang, 2017), studies investigating the association between domain-general cognitive skills and word recognition have been limited. Through conducting a multigroup comparison, the model of EF’s direct and indirect effects on word recognition was found to hold true for both native and CSL learners, echoing the “universals of learning to read” proposed by Perfetti and his colleagues (e.g., Perfetti & Harris, 2013; Verhoeven & Perfetti, 2022). As demonstrated by Verhoeven and Perfetti (2022), despite specific variations in the form-sound-meaning relationships across languages (such as Chinese radicals providing both semantic and phonological clues, while English letters represent phonemes only), there is a presumed universality in cognitive skills such as EF in addressing the common challenge of establishing the form-sound-meaning mapping. By involving L2 learners, the present findings further provide empirical evidence for the critical role of EF in supporting reading, regardless of distinctions among different learning groups.

More importantly, the present study is perhaps the first to compare the effect of EF on word recognition between native and CSL learners. The indirect effect of EF on word recognition via orthographic knowledge was significantly stronger among L2 learners than among their native counterparts. Research on L2 language processing has revealed that both L1 and L2 words are activated during L2 reading (e.g., Bergmann et al., 2015; Gastmann & Poarch, 2022; Hopp, 2017), even if there are significant differences in scripts between the reader’s L1 and L2 (e.g., Kroll et al., 2014). Therefore, L2 learners may have more cognitive burden during L2 reading from managing between languages. Previous studies have identified that cognitive factors such as EF are critical to language selection and use among L2 learners (e.g., Green & Abutalebi, 2013; Luque & Morgan-Short, 2021). The stronger indirect effect of EF on L2 word recognition implies the importance of EF in effectively managing language repertoires at the lexical level. Specifically, given that both languages are activated during reading, L2 learners may employ more EF to apply orthographic rules of a specific language and inhibit interference from their L1. Additionally, the CSL learners who participated in our study mostly have alphabetic languages as their first language. Previous studies have reported that, unlike their native Chinese counterparts who perceive Chinese characters as a whole, they tend to perceive the components of Chinese characters in a horizontal way due to their habit of reading alphabetic languages, where letters are presented in a linear sequence (Loh et al., 2021). Therefore, CSL learners may depend more heavily on EF to regulate this visual habit, thereby enhancing their ability to recognize Chinese characters.

Furthermore, we could refer to evidence regarding students’ lexical processing in word recognition. Previous studies using prime-target tasks have reported that characters with identical radicals are more easily activated among L2 learners than native learners (e.g., Taft & Li, 2020; Jiang & Wu, 2022; Qiao & Forster, 2017). Specifically, for native learners, only orthographically similar words that were semantically related were activated (e.g., Jiang, 2021; Qiao & Forster, 2017), whereas no restrictions were found in the activation effect among L2 learners (e.g., Taft & Li, 2020; Jiang & Wu, 2022). Given that characters with identical radicals are more easily activated, it is not surprising that CSL learners require more EF to coordinate orthographic knowledge during word recognition. CSL learners may need EF to switch quickly between the simultaneously activated options and inhibit the irrelevant characters. Moreover, EF is needed to hold the visual input in working memory to accurately compare and distinguish it from the orthographic form stored in their long-term memory.

## Limitations and Implications

There are a few limitations of the present study. First, we did not include the indirect path via phonological awareness in the present study. Previous studies have suggested that phonological awareness may develop later than other metalinguistic skills such as orthographic awareness and morphological awareness, particularly among L2 learners (e.g., Berninger et al., 2010; Zhao et al., 2017). Moreover, CSL learners’ L1 differs significantly from Cantonese in terms of tone system, rhyme system, and syllable structure. For example, their L1s are mostly non-tonal languages or languages with very simple tone systems. Considering the potential floor effect that may be found among CSL learners in phonological tasks, phonological awareness was not included. Further studies are needed to compare the similarities and differences in the interaction between EF and phonological awareness in word recognition between native and CSL learners. Second, the present study utilized a morphological recognition task and a morphological discrimination task to measure morphological awareness, while there were studies employed a series of morphological awareness tasks (e.g., McBride-Chang et al., 2003). To gain a more comprehensive understanding of the specific associations between EF, morphological awareness, and word reading, future studies should consider evaluating morphological awareness in a more detailed manner. Third, due to the great typological differences between CSL learners’ native language and Chinese (Brothers et al., 2021), the ability of CSL learners’ native language was not measured in the present study. Future studies can address this issue to further understand how the level of L1 affects the function of EF in L2 learners’ word recognition performance.

Additionally, the present study was conducted in the context of traditional Chinese. Previous studies have suggested that the recognition of traditional Chinese and simplified Chinese could be different due to the differences between the two writing systems (e.g., Liu & Hsiao, 2012). For example, the written form of traditional Chinese is visually more complex than simplified Chinese (e.g., 礼 *gift* is written as 禮 in traditional Chinese). Thus, the generalization of our findings to Simplified Chinese learners should be made with caution.

Despite the limitations, our findings could shed some light on pedagogy. In the present study, the prominent direct effect of EF underscores the importance of EF in supporting word recognition, which can extend from kindergarten to primary school learning stages, as suggested by previous longitudinal studies (e.g., Ren et al., 2022). Although EF can be enhanced with training, the effectiveness of applying these practiced EF skills in real-world contexts, such as learning literacy skills, can be affected by the paradigms of the EF training (Takacs & Kassai, 2019). Specifically, compared with explicit training on EF skills, tasks that foster EF skills implicitly seem to be more effective. Tasks such as biofeedback-enhanced relaxation, strategy teaching programs, and mindfulness practices have been found to be effective approaches in implicitly enhancing learners’ EF skills, as suggested by Takacs and Kassai (2019). These approaches could be considered by teachers and parents to better assist learners in enhancing their EF skills.

In addition, the significant indirect effect from EF to word reading via orthographic knowledge implies the challenge of employing orthographic awareness in word recognition, particularly among CSL learners. Previous studies have proposed the importance of explicit instruction on orthographic knowledge for both native and CSL learners (e.g., Ke, 2020). The present findings further suggest the importance of teaching the application of orthographic knowledge. For example, after teaching a radical, the teacher can provide some unlearned words for the students to recognize, thereby strengthening the students’ ability to use the radical knowledge they have learned. Additionally, teachers could present irregular characters that contain learned radicals and explain the inconsistency between the character and the radical, encouraging students to be more careful in using their orthographic knowledge during word recognition.

## References

[CR132] Aboud, K. S., Bailey, S. K., Petrill, S. A., & Cutting, L. E. (2016). Comprehending text versus reading words in young readers with varying reading ability: Distinct patterns of functional connectivity from common processing hubs. *Developmental Science,**19*(4), 632–656. 27147257 10.1111/desc.12422 PMC4945471

[CR1] Altani, A., Protopapas, A., & Georgiou, G. K. (2017). The contribution of executive functions to naming digits, objects, and words. *Reading and Writing,**30*(1), 121–141.

[CR125] Baddeley, A. D., & Hitch, G. J. (1974). Working memory. In G. H. Bower (Ed.), *The psychology of learning and motivation* (Vol. 8, pp. 47–89). New York: Academic Press.

[CR2] Bergmann, C., Sprenger, S. A., & Schmid, M. S. (2015). The impact of language co-activation on L1 and L2 speech fluency. *Acta Psychologica,**161*, 25–35. 26298087 10.1016/j.actpsy.2015.07.015

[CR3] Bialystok, E., Barac, R., Blaye, A., & Poulin-Dubois, D. (2010). Word mapping and executive functioning in young monolingual and bilingual children. *Journal of Cognition and Development,**11*(4), 485–508. 21197133 10.1080/15248372.2010.516420 PMC3010730

[CR4] Bialystok, E., & Martin, M. M. (2003). Notation to symbol: Development in children’s understanding of print. *Journal of Experimental Child Psychology,**86*(3), 223–243. 14559205 10.1016/s0022-0965(03)00138-3

[CR126] Brocki, K. C., & Tillman, C. (2014). Mental set shifting in childhood: The role of working memory and inhibitory control. *Infant and Child Development,**23*(6), 588–604.

[CR5] Brothers, T., Hoversten, L. J., & Traxler, M. J. (2021). Bilinguals on the garden-path: Individual differences in syntactic ambiguity resolution. *Bilingualism: Language and Cognition,**24*(4), 612–627. 10.1017/S1366728920000711 35669170 10.1017/s1366728920000711 PMC9164278

[CR130] Brydges, C. R., Fox, A. M., Reid, C. L., & Anderson, M. (2014). The differentiation of executive functions in middle and late childhood: A longitudinal latent-variable analysis. *Intelligence,**47*, 34–43.

[CR6] Burgoyne, K., Malone, S., Lervag, A., & Hulme, C. (2019). Pattern understanding is a predictor of early reading and arithmetic skills. *Early Childhood Research Quarterly,**49*, 69–80.

[CR201] Cai, M., & Liao, X. (2024). The relationship between vocabulary depth knowledge, word reading, and reading comprehension in Chinese. *Journal of Experimental Child Psychology, 244*, 105951. 38735223 10.1016/j.jecp.2024.105951

[CR7] Cartwright, K. B., Bock, A. M., Clause, J. H., August, E. A. C., Saunders, H. G., & Schmidt, K. J. (2020). Near-and far-transfer effects of an executive function intervention for 2nd to 5th-grade struggling readers. *Cognitive Development,**56*, 100932.

[CR8] Cartwright, K. B., Marshall, T. R., Huemer, C. M., & Payne, J. B. (2019). Executive function in the classroom: Cognitive flexibility supports reading fluency for typical readers and teacher-identified low-achieving readers. *Research in Developmental Disabilities,**88*, 42–52. 30851482 10.1016/j.ridd.2019.01.011

[CR9] Chan, T. S., Loh, E. K., & Hung, C. O. (2021). A longitudinal study of Chinese as a second language kindergarteners’ orthographic awareness and its association with their lexical learning performance. *Current Psychology,**42*, 1–12.33519148

[CR142] Chang, C. L. (2009). Exploring the results of foreign students’ reading and writing capacities taught by the strategy differentiating chinese character range between reading and writing teaching experiments based on the foreign beginners without knowledge of Chinese characters. *Chung Yuan CSL Bulletin,**3*, 55–74.

[CR10] Chelune, G. J., & Baer, R. A. (1986). Developmental norms for the wisconsin card sorting test. *Journal of Clinical and Experimental Neuropsychology,**8*(3), 219–228. 3722348 10.1080/01688638608401314

[CR11] Chen, A. S. W. (2021). Form-first approach in foreign language word learning. *Second Language Research,**37*(1), 51–68.

[CR12] Chen, Y. C., & Yeh, S. L. (2015). Binding radicals in Chinese character recognition: Evidence from repetition blindness. *Journal of Memory and Language,**78*, 47–63.

[CR13] Chen, Xi., & Zhao, J. (2022). Reading-related skills associated with acquisition of Chinese as a second/foreign language: a meta-analysis. *Frontiers in Psychology*. 10.3389/fpsyg.2022.783964 35369154 10.3389/fpsyg.2022.783964 PMC8966685

[CR14] Choi, W., Tong, X., Law, K. K. S., & Cain, K. (2018). Within-and cross-language contributions of morphological awareness to word reading development in Chinese-English bilingual children. *Reading and Writing,**31*(8), 1787–1820.

[CR15] Chung, K. K. H., Lam, C. B., & Cheung, K. C. (2018). Visuomotor integration and executive functioning are uniquely linked to Chinese word reading and writing in kindergarten children. *Reading and Writing,**31*(1), 155–171.

[CR16] Chung, F. H. K., & Leung, M. T. (2018). Data analysis of Chinese characters in primary school corpora of Hong Kong and mainland China: Preliminary theoretical interpretations. *Clinical Linguistics & Phonetics,**22*(4–5), 379–389. 10.1080/02699200701776757 18415738

[CR17] Chung, K. K., & McBride-Chang, C. (2011). Executive functioning skills uniquely predict Chinese word reading. *Journal of Educational Psychology,**103*(4), 909.

[CR18] Coltheart, M., Rastle, K., Perry, C., Langdon, R., & Ziegler, J. (2001). DRC: A dual route cascaded model of visual word recognition and reading aloud. *Psychological Review,**108*(1), 204. 11212628 10.1037/0033-295x.108.1.204

[CR19] Colé, P., Duncan, L. G., & Blaye, A. (2014). Cognitive flexibility predicts early reading skills. *Frontiers in Psychology,**5*, 565. 24966842 10.3389/fpsyg.2014.00565 PMC4052802

[CR20] Davis, J. N., & Bistodeau, L. (1993). How do L1 and L2 reading differ? Evidence from think aloud protocols. *The Modern Language Journal,**77*(4), 459–472.

[CR146] Derwing, T. M., & Munro, M. J. (2013). The development of L2 oral language skills in two L1 groups: A 7-year study. *Language Learning,**63*(2), 163–185.

[CR21] Desrochers, A., Manolitsis, G., Gaudreau, P., & Georgiou, G. (2018). Early contribution of morphological awareness to literacy skills across languages varying in orthographic consistency. *Reading and Writing,**31*, 1695–1719.

[CR22] Diamond, A. (2013). Executive functions. *Annual Review of Psychology,**64*, 135. 23020641 10.1146/annurev-psych-113011-143750 PMC4084861

[CR23] Ding, G., Peng, D., & Taft, M. (2004). The nature of the mental representation of radicals in Chinese: A priming study. *Journal of Experimental Psychology: Learning, Memory, and Cognition,**30*(2), 530. 14979822 10.1037/0278-7393.30.2.530

[CR25] Fabrigar, L. R., Wegener, D. T., MacCallum, R. C., & Strahan, E. J. (1999). Evaluating the use of exploratory factor analysis in psychological research. *Psychological Methods,**4*(3), 272.

[CR26] Ferrand, L., & Grainger, J. (2003). Homophone interference effects in visual word recognition. *The Quarterly Journal of Experimental Psychology Section A,**56*(3), 403–419. 10.1080/02724980244000422 12745841

[CR27] Fong, C. Y., & Chung, P. Y. (2020). The role of orthographic flexibility in Chinese word reading among kindergarten children. *Educational Psychology,**40*(7), 804–819.

[CR28] Fong, C. Y., & Ho, C. S. H. (2022). Executive functions in Chinese kindergarten children with early reading problems. *Dyslexia,**28*(3), 325–341. 10.1002/dys.1714 35586880 10.1002/dys.1714

[CR29] Fuhs, M. W., Nesbitt, K. T., Farran, D. C., & Dong, N. (2014). Longitudinal associations between executive functioning and academic skills across content areas. *Developmental Psychology,**50*, 1698–1709. 10.1037/a0036633 24749550 10.1037/a0036633

[CR145] Gao, F., & Shum, M. S. (2010). Investigating the role of bilingual teaching assistants in Hong Kong: An exploratory study. *Educational Research,**52*(4), 445–456.

[CR30] Gao, F. A. N. G., & Gube, J. (2020). Multicultural education: How are ethnic minorities labelled and educated in post-handover Hong Kong. *Migration and Language Education,**2*(3), 51–59.

[CR31] Gastmann, F., & Poarch, G. J. (2022). Cross-language activation during word recognition in child second-language learners and the role of executive function. *Journal of Experimental Child Psychology,**221*, 105443. 35623309 10.1016/j.jecp.2022.105443

[CR32] Giazitzidou, S., Mouzaki, A., & Padeliadu, S. (2023). Pathways from morphological awareness to reading fluency: The mediating role of phonological awareness and vocabulary. *Reading and Writing,**37*, 1–23.

[CR33] Gong, Y., Lai, C., & Gao, X. (2020). The teaching and learning of Chinese as a second or foreign language: The current situation and future directions. *Frontiers of Education in China,**15*(1), 1–13.

[CR34] Grant, D. A., & Berg, E. (1948). A behavioral analysis of degree of reinforcement and ease of shifting to new responses in a Weigl-type card-sorting problem. *Journal of Experimental Psychology.,**38*(4), 404–411. 18874598 10.1037/h0059831

[CR35] Grant, A. M., Fang, S. Y., & Li, P. (2015). Second language lexical development and cognitive control: A longitudinal fMRI study. Brain and language, 144, 35–47. Grant, A. M., Fang, S. Y., & Li, P. (2015). Second language lexical development and cognitive control: A longitudinal fMRI study. *Brain and Language,**144*, 35–47. 25899988 10.1016/j.bandl.2015.03.010

[CR36] Green, D. W., & Abutalebi, J. (2013). Language control in bilinguals: The adaptive control hypothesis. *Journal of Cognitive Psychology,**25*(5), 515–530. 25077013 10.1080/20445911.2013.796377 PMC4095950

[CR37] Gu, J., Zhou, J., Bao, Y., Liu, J., Perea, M., & Li, X. (2022). The effect of transposed-character distance in Chinese reading. *Journal of Experimental Psychology: Learning, Memory, and Cognition.*10.1037/xlm0001180 36037495 10.1037/xlm0001180

[CR38] Hamada, M., & Koda, K. (2011). Similarity and difference in learning L2 word-form. *System,**39*(4), 500–509.

[CR39] Hamilton, S., Freed, E., & Long, D. L. (2016). Word-decoding skill interacts with working memory capacity to influence inference generation during reading. *Reading Research Quarterly,**51*(4), 391–402. 27833213 10.1002/rrq.148 PMC5098811

[CR40] Ho, C. S. H., Ng, T. T., & Ng, W. K. (2003). A “radical” approach to reading development in Chinese: The role of semantic radicals and phonetic radicals. *Journal of Literacy Research,**35*(3), 849–878.

[CR41] Hopp, H. (2017). Cross-linguistic lexical and syntactic co-activation in L2 sentence processing. *Linguistic Approaches to Bilingualism,**7*(1), 96–130.

[CR42] Hu, L. T., & Bentler, P. M. (1999). Cutoff criteria for fit indexes in covariance structure analysis: Conventional criteria versus new alternatives. *Structural Equation Modeling: A Multidisciplinary Journal,**6*(1), 1–55.

[CR43] Huo, S., Zhang, X., & Law, Y. K. (2021). Pathways to word reading and calculation skills in young Chinese children: From biologically primary skills to biologically secondary skills. *Journal of Educational Psychology,**113*(2), 230.

[CR44] Inoue, T., Zhang, S. Z., & Georgiou, G. K. (2022). Direct and indirect effects of cognitive-linguistic and home environment factors on pinyin reading development. *Educational Psychology,**42*, 1–17.

[CR45] Jiang, N. (2021). Orthographic friends and lexical strangers in the L2 lexicon. *Journal of Second Language Studies,**4*, 224–244. 10.1075/jsls.21012.jia

[CR46] Jiang, N., Hou, F., & Jiang, X. (2020). Analytic versus holistic recognition of Chinese words among L2 learners. *The Modern Language Journal,**104*(3), 567–580.

[CR47] Jiang, N., & Wu, X. (2022). Orthographic priming in second-language visual word recognition. *Language Learning,**72*(3), 625–645.

[CR135] Jiang, N. (2018). *Second language processing: An introduction*. London: Routledge.

[CR48] Jiang, N., & Zhang, J. (2021). Form prominence in the L2 lexicon: Further evidence from word association. *Second Language Research,**37*(1), 69–90.

[CR49] Karr, J. E., Areshenkoff, C. N., Rast, P., Hofer, S. M., Iverson, G. L., & Garcia-Barrera, M. A. (2018). The unity and diversity of executive functions: A systematic review and re-analysis of latent variable studies. *Psychological Bulletin,**144*(11), 1147. 30080055 10.1037/bul0000160 PMC6197939

[CR50] Kaushanskaya, M., Park, J. S., Gangopadhyay, I., Davidson, M. M., & Weismer, S. E. (2017). The relationship between executive functions and language abilities in children: A latent variables approach. *Journal of Speech, Language, and Hearing Research,**60*(4), 912–923. 28306755 10.1044/2016_JSLHR-L-15-0310 PMC5548084

[CR149] Ke, S. E. (2020). Review of research on learning and instruction with specific reference to reading Chinese as an additional language (1976–2018). *Frontiers of Education in China,**15*(1), 14–38.

[CR51] Kieffer, M. J., Vukovic, R. K., & Berry, D. (2013). Roles of attention shifting and inhibitory control in fourth-grade reading comprehension. *Reading Research Quarterly,**48*(4), 333–348.

[CR52] Kim, Y. S. G. (2017). Why the simple view of reading is not simplistic: Unpacking component skills of reading using a direct and indirect effect model of reading (DIER). *Scientific Studies of Reading,**21*(4), 310–333.

[CR53] Kim, Y. S. G. (2020). Hierarchical and dynamic relations of language and cognitive skills to reading comprehension: Testing the direct and indirect effects model of reading (DIER). *Journal of Educational Psychology,**112*(4), 667.

[CR54] Kim, S. A., Christianson, K., & Packard, J. (2015). Working memory in L2 character processing: The case of learning to read Chinese. *Working Memory in Second Language Acquisition and Processing. Bristol: Multilingual Matters,**87*, 85–104.

[CR55] Kline, T. (2005). *Psychological testing: A practical approach to design and evaluation*. Thousand Oaks: Sage.

[CR147] van Koert, M., Leona, N., Rispens, J., Tijms, J., Molen, M. V. D., Grunberg, H. L., & Snellings, P. (2023). English Grammar Skills in Dutch Grade 4 Children: Examining the Relation Between L1 and L2 Language Skills. *Journal of Psycholinguistic Research,**52*(5), 1737–1753. 37184734 10.1007/s10936-023-09968-x PMC10520197

[CR56] Koda, K. (2021). Transfer facilitation effects of morphological awareness on multicharacter word reading in Chinese as a foreign language. *Applied Psycholinguistics,**42*(5), 1263–1286.

[CR134] Kroll, J. F., & Stewart, E. (1994). Category interference in translation and picture naming: Evidence for asymmetric connections between bilingual memory representations. *Journal of Memory and Language,**33*(2), 149–174.

[CR57] Kroll, J. F., Bobb, S. C., & Hoshino, N. (2014). Two languages in mind: Bilingualism as a tool to investigate language, cognition, and the brain. *Current Directions in Psychological Science,**23*(3), 159–163. 25309055 10.1177/0963721414528511 PMC4191972

[CR58] Ku, Y. M., & Anderson, R. C. (2003). Development of morphological awareness in Chinese and english. *Reading and Writing,**16*(5), 399–422.

[CR139] Ku, H. B., Chan, K. W., & Sandhu, K. K. (2005). *Education of South Asian ethnic minority groups in Hong Kong*. Hong Kong: Centre for Social Policy Studies, The Hong Kong Polytechnic University.

[CR59] Leong, C. K., Tse, S. K., Loh, K. Y., & Ki, W. W. (2011). Orthographic knowledge important in comprehending elementary Chinese text by users of alphasyllabaries. *Reading Psychology,**32*(3), 237–271.

[CR60] Li, M., Jiang, N., & Gor, K. (2017). L1 and L2 processing of compound words: Evidence from masked priming experiments in English. *Bilingualism: Language and Cognition,**20*, 384–402. 10.1017/S1366728915000681

[CR61] Li, X., & Koda, K. (2022). Linguistic constraints on the cross-linguistic variations in L2 word recognition. *Reading and Writing,**35*(6), 1401–1424.

[CR62] Li, H., Shu, H., McBride-Chang, C., Liu, H., & Peng, H. (2012). Chinese children’s character recognition: Visuo-orthographic, phonological processing and morphological skills. *Journal of Research in Reading,**35*(3), 287–307.

[CR124] Li, H., & Rao, N. (2000). Parental influences on Chinese literacy development: A comparison of preschoolers in Beijing, Hong Kong, and Singapore. *International Journal of Behavioral Development,**24*(1), 82–90.

[CR200] Liao, X., Loh, E. K. Y., & Cai, M. (2022). Lexical orthographic knowledge mediates the relationship between character reading and reading comprehension among learners with Chinese as a second language. *Frontiers in Psychology, 13*, 779905. 35432117 10.3389/fpsyg.2022.779905 PMC9009092

[CR63] Liu, C., & Chung, K. K. H. (2022). Bidirectional relations among paired associate learning, language-specific skills and chinese word reading in kindergarten children. *Early Education and Development,**33*(4), 723–738.

[CR64] Liu, C., Chung, K. K. H., & Fung, W. K. (2019). Bidirectional relationships between children’s executive functioning, visual skills, and word reading ability during the transition from kindergarten to primary school. *Contemporary Educational Psychology,**59*, 101779.

[CR65] Liu, D., Li, H., & Wong, K. S. R. (2017). The anatomy of the role of morphological awareness in Chinese character learning: The mediation of vocabulary and semantic radical knowledge and the moderation of morpheme family size. *Scientific Studies of Reading,**21*(3), 210–224.

[CR66] Liu, P. D., McBride-Chang, C., Wong, T. T. Y., Shu, H. U. A., & Wong, A. M. Y. (2013). Morphological awareness in Chinese: Unique associations of homophone awareness and lexical compounding to word reading and vocabulary knowledge in Chinese children. *Applied Psycholinguistics,**34*(4), 755–775.

[CR67] Liu, Y., Zhang, S., Zhang, Y., Diao, J., Cheng, Q., Gao, R., & Mo, L. (2023). Study of the orthographic neighborhood frequency effect on Chinese compound characters. *Reading and Writing,**36*(10), 2717–2738. 10.1007/s11145-022-10392-1

[CR143] Loh, E. K., Tam, L. C., Lau, C. P., & Leung, S. O. (2017). How ethnic minority students perceive patterns in Chinese characters: Knowledge of character components and structures. *Chinese as a Second Language Assessment*. 10.1007/978-981-10-4089-4_5

[CR68] Loh, E. K. Y., Liao, X., & Leung, S. O. (2018). Acquisition of orthographic knowledge: Developmental difference among learners with Chinese as a second language (CSL). *System,**74*, 206–216.

[CR69] Loh, E. K. Y., Liao, X., & Leung, S. O. (2021). How do Chinese as a second language (CSL) learners acquire orthographic knowledge: component, structure and position regularity. *Language awareness,**30*, 297–316.

[CR141] Loh, E. K. Y., Mak, T. F. M., & Tam, C. W. L. (2015). *The road to successful Chinese language learning: Effective strategies for teaching and learning Chinese characters. Infusing IB philosophy and pedagogy into Chinese language teaching*. Woodbridge: John Catt Educational Ltd.

[CR70] Luque, A., & Morgan-Short, K. (2021). The relationship between cognitive control and second language proficiency. *Journal of Neurolinguistics,**57*, 100956.

[CR71] Ma, X., Gong, Y., Gao, X., & Xiang, Y. (2017). The teaching of Chinese as a second or foreign language: A systematic review of the literature 2005–2015. *Journal of Multilingual and Multicultural Development,**38*(9), 815–830.

[CR72] McBride, C. A. (2016). Is Chinese special? Four aspects of Chinese literacy acquisition that might distinguish learning Chinese from learning alphabetic orthographies. *Educational Psychology Review,**28*(3), 523–549.

[CR73] McBride-Chang, C., Shu, H., Zhou, A., Wat, C. P., & Wagner, R. K. (2003). Morphological awareness uniquely predicts young children’s Chinese character recognition. *Journal of Educational Psychology,**95*(4), 743.

[CR127] Miyake, A., Friedman, N. P., Emerson, M. J., Witzki, A. H., Howerter, A., & Wager, T. D. (2000). The unity and diversity of executive functions and their contributions to complex “frontal lobe” tasks: A latent variable analysis. *Cognitive Psychology,**41*(1), 49–100. 10945922 10.1006/cogp.1999.0734

[CR74] Miyake, A., & Friedman, N. P. (2012). The nature and organization of individual differences in executive functions: Four general conclusions. *Current Directions in Psychological Science,**21*(1), 8–14. 22773897 10.1177/0963721411429458 PMC3388901

[CR75] Moon, J., & Jiang, N. (2012). Non-selective lexical access in different-script bilinguals. *Bilingualism: Language and Cognition,**15*(1), 173–180.

[CR76] Nassaji, H. (2014). The role and importance of lower-level processes in second language reading. *Language Teaching,**47*, 1–37. 10.1017/S0261444813000396

[CR77] Newman, S. D. (2012). The homophone effect during visual word recognition in children: An fMRI study. *Psychological Research Psychologische Forschung,**76*(3), 280–291. 21660483 10.1007/s00426-011-0347-2

[CR78] Ober, T. M., Brooks, P. J., Plass, J. L., & Homer, B. D. (2019). Distinguishing direct and indirect effects of executive functions on reading comprehension in adolescents. *Reading Psychology,**40*(6), 551–581.

[CR79] Packard, J. L. (2000). *The morphology of Chinese: A linguistic and cognitive approach*. Cambridge: Cambridge University Press.

[CR80] Pan, D. J., & Lin, D. (2023). How do executive functions explain early Chinese reading and writing? *Reading and Writing,**36*(3), 625–647.

[CR81] Pan, D. J., Yang, X., Lui, K. F. H., Lo, J. C. M., McBride, C., & Ho, C. S. H. (2021). Character and word reading in Chinese: Why and how they should be considered uniquely vis-a-vis literacy development. *Contemporary Educational Psychology,**65*, 101961.

[CR82] Peng, P., Barnes, M., Wang, C. C., Wang, W., Li, S., Lee Swanson, H., Dardick, W., & Tao, S. (2018). Meta-analysis on the relation between reading and working memory. *Psychological Bulletin,**144*(1), 48–76. 10.1037/bul0000124 29083201 10.1037/bul0000124

[CR83] Perfetti, C. (2007). Reading ability: Lexical quality to comprehension. *Scientific Studies of Reading,**11*(4), 357–383.

[CR84] Perfetti, C. A., & Harris, L. N. (2013). Universal reading processes are modulated by language and writing system. *Language Learning and Development,**9*(4), 296–316.

[CR85] Perfetti, C. A., Liu, Y., & Tan, L. H. (2005). The lexical constituency model: Some implications of research on Chinese for general theories of reading. *Psychological Review,**112*(1), 43. 15631587 10.1037/0033-295X.112.1.43

[CR86] Perikova, E. I., Filippova, M. G., Makarova, D. N., & Gnedykh, D. S. (2024). The benefits of labeling in fast mapping and explicit encoding. *Neuroscience and Behavioral Physiology,**54*(3), 424–433. 10.1007/s11055-024-01609-7

[CR133] Poarch, G. J., & Van Hell, J. G. (2012). Executive functions and inhibitory control in multilingual children: Evidence from second-language learners, bilinguals, and trilinguals. *Journal of Experimental Child Psychology,**113*(4), 535–551. 22892367 10.1016/j.jecp.2012.06.013

[CR87] Protopapas, A., Archonti, A., & Skaloumbakas, C. (2007). Reading ability is negatively related to Stroop interference. *Cognitive Psychology,**54*(3), 251–282. 16962090 10.1016/j.cogpsych.2006.07.003

[CR88] Putnick, D. L., & Bornstein, M. H. (2016). Measurement invariance conventions and reporting: The state of the art and future directions for psychological research. *Developmental Review,**41*, 71–90. 27942093 10.1016/j.dr.2016.06.004 PMC5145197

[CR89] Qiao, X., & Forster, K. I. (2017). Is the L2 lexicon different from the L1 lexicon? Evidence from novel word lexicalization. *Cognition,**158*, 147–152. 10.1016/j.cognition.2016.10.026 27835785 10.1016/j.cognition.2016.10.026

[CR90] Raudszus, H., Segers, E., & Verhoeven, L. (2019). Situation model building ability uniquely predicts first and second language reading comprehension. *Journal of Neurolinguistics,**50*, 106–119.

[CR91] Ren, L., Hu, B. Y., & Wu, H. (2022). Early executive function predicts children’s Chinese word reading from preschool through grade 3. *Contemporary Educational Psychology,**69*, 102054.

[CR92] Schmitt, S. A., Geldhof, G. J., Purpura, D. J., Duncan, R., & McClelland, M. M. (2017). Examining the relations between executive function, math, and literacy during the transition to kindergarten: A multi-analytic approach. *Journal of Educational Psychology,**109*(8), 1120–1140. 10.1037/edu0000193

[CR93] Seidenberg, M. S. (2011). Reading in different writing systems: One architecture, multiple solutions. In P. McCardle, B. Miller, J. R. Lee, & O. J. L. Tzeng (Eds.), *Dyslexia across languages: Orthography and the brain–gene–behavior link* (pp. 146–168). Paul H Brookes Publishing.

[CR94] Shu, H., McBride-Chang, C., Wu, S., & Liu, H. (2006). Understanding Chinese developmental dyslexia: Morphological awareness as a core cognitive construct. *Journal of Educational Psychology,**98*(1), 122.

[CR131] Spencer, M., Richmond, M. C., & Cutting, L. E. (2020). Considering the role of executive function in reading comprehension: A structural equation modeling approach. *Scientific Studies of Reading,**24*(3), 179–199. 32982142 10.1080/10888438.2019.1643868 PMC7518696

[CR96] Spencer, M., & Cutting, L. E. (2021). Relations among executive function, decoding, and reading comprehension: An investigation of sex differences. *Discourse Processes,**58*(1), 42–59. 33716362 10.1080/0163853X.2020.1734416 PMC7954233

[CR128] St Clair-Thompson, H. L., & Gathercole, S. E. (2006). Executive functions and achievements in school: Shifting, updating, inhibition, and working memory. *Quarterly Journal of Experimental Psychology,**59*(4), 745–759. 10.1080/17470210500162854 16707360

[CR97] Sun, J., Pae, H. K., & Ai, H. (2021). The recognition of coordinative compound words by learners of Chinese as a foreign language: A mixed methods study. *Foreign Language Annals,**54*(4), 923–951.

[CR98] Tabachnick, B. G., & Fidell, L. S. (2001). *Using multivariate statistics*. Boston: Allyn & Bacon.

[CR99] Taboada Barber, A., Cartwright, K. B., Hancock, G. R., & Klauda, S. L. (2021). Beyond the simple view of reading: The role of executive functions in emergent bilinguals’ and english monolinguals’ reading comprehension. *Reading Research Quarterly,**56*, S45–S64.

[CR148] Takacs, Z. K., & Kassai, R. (2019). The efficacy of different interventions to foster children’s executive function skills: a series of meta-analyses. *Psychological Bulletin,**145*(7), 653. 31033315 10.1037/bul0000195

[CR100] Taft, M., & Li, J. (2021). A new type of masked form priming: Native versus nonnative English speakers. *Studies in Second Language Acquisition,**43*(2), 442–453.

[CR101] Taft, M. (2006). Processing of characters by native Chinese readers. In P. Li, L. H. Tan, E. Bates, & O. J. L. Tzeng (Eds.), *Handbook of East Asian Psycholinguistics. *Cambridge: Cambridge University Press.

[CR102] Tong, X., Chung, K. K. H., & McBride, C. (2014). Two-character Chinese compound word processing in Chinese children with and without dyslexia: ERP evidence. *Developmental Neuropsychology,**39*(4), 285–301. 24854773 10.1080/87565641.2014.907720

[CR103] Tong, X., Leung, W. W. S., & Tong, X. (2019). Visual statistical learning and orthographic awareness in Chinese children with and without developmental dyslexia. *Research in Developmental Disabilities,**92*, 103443. 31374382 10.1016/j.ridd.2019.103443

[CR104] Tong, X., Tong, X., & McBride, C. (2017). Unpacking the relation between morphological awareness and Chinese word reading: Levels of morphological awareness and vocabulary. *Contemporary Educational Psychology,**48*, 167–178.

[CR105] Vales, C., & Fisher, A. V. (2019). When stronger knowledge slows you down: Semantic relatedness predicts children’s co-activation of related items in a visual search paradigm. *Cognitive Science,**43*(6), e12746. 31204802 10.1111/cogs.12746

[CR110] Van de Weijer-Bergsma, E., Kroesbergen, E. H., Prast, E. J., & Van Luit, J. E. (2015). Validity and reliability of an online visual–spatial working memory task for self-reliant administration in school-aged children. *Behavior Research Methods,**47*(3), 708–719. 24771322 10.3758/s13428-014-0469-8

[CR24] Van Der Elst, W. I. M., Van Boxtel, M. P., Van Breukelen, G. J., & Jolles, J. (2008). Detecting the significance of changes in performance on the Stroop color-word test, rey’s verbal learning test, and the letter digit substitution test: The regression-based change approach. *Journal of the International Neuropsychological Society,**14*(1), 71–80. 18078533 10.1017/S1355617708080028

[CR95] Van der Sluis, S., De Jong, P. F., & Van der Leij, A. (2007). Executive functioning in children, and its relations with reasoning, reading, and arithmetic. *Intelligence,**35*(5), 427–449.

[CR106] Verhoeven, L., & Perfetti, C. (2022). Universals in learning to read across languages and writing systems. *Scientific Studies of Reading,**26*(2), 150–164.

[CR107] Wang, Y., & McBride, C. (2016). Character reading and word reading in Chinese: Unique correlates for Chinese kindergarteners. *Applied Psycholinguistics,**37*(2), 371–386.

[CR144] Wang, D. (2021). *Teaching Chinese as a second language to ethnic minority learners in Hong Kong Multilingual China*. London: Routledge.

[CR108] Wechsler, D. (2003). *WISC-IV technical and interpretive manual San Antonio*. Psychological Corporation.

[CR109] Wei, J. (2012). *The effect of phonological working memory on children's Chinese spoken word learning* (Doctoral dissertation, University of Illinois at Urbana-Champaign).

[CR111] Wong, Y. K. (2017). The role of radical awareness in Chinese-as-a-second-language learners’ Chinese character reading development. *Language Awareness,**26*(3), 211–225.

[CR112] Wong, Y. K. (2018). Structural relationships between second-language future self-image and the reading achievement of young Chinese language learners in Hong Kong. *System,**72*, 201–214.

[CR113] Wong, Y. K., & Shiu, L. P. (2009). Chinese language attainment of ethnic minority primary school students. *Journal of Basic Education,**18*(2), 123–136.

[CR114] Xu, Y., Chang, L. Y., & Perfetti, C. A. (2014). The effect of radical-based grouping in character learning in Chinese as a foreign language. *The Modern Language Journal,**98*(3), 773–793.

[CR115] Yang, X., Peng, P., & Meng, X. (2019). How do metalinguistic awareness, working memory, reasoning, and inhibition contribute to Chinese character reading of kindergarten children? *Infant and Child Development,**28*(3), e2122.

[CR116] Yang, X., & Qiao, L. (2021). Direct effects of visual skills and working memory on Chinese character reading in young children. *Infant and Child Development,**30*(4), e2231.

[CR117] ZHOU, Y., & McBRIDE-CHANG, C. (2013, July). Chinese and English word reading in 8 and 9 year old children learning Chinese as a foreign language in Hong Kong. In *Twentieth Annual Meeting of Society for the Scientific Study of Reading*.

[CR136] Yeung, P. S., Ho, C. S. H., Chik, P. P. M., Lo, L. Y., Luan, H., Chan, D. W. O., & Chung, K. K. H. (2011). Reading and spelling Chinese among beginning readers: What skills make a difference? *Scientific Studies of Reading,**15*(4), 285–313.

[CR137] Yeung, P. S., Ho, C. S. H., Wong, Y. K., Chan, D. W. O., Chung, K. K. H., & Lo, L. Y. (2013). Longitudinal predictors of Chinese word reading and spelling among elementary grade students. *Applied Psycholinguistics,**34*(6), 1245–1277.

[CR118] Zhang, D. (2017). Word reading in L1 and L2 learners of Chinese: Similarities and differences in the functioning of component processes. *The Modern Language Journal,**101*(2), 391–411.

[CR138] Zhang, Q., Tsung, L., Cruickshank, K., Ki, W. W., & Shum, M. S. K. (2010). South Asian students’ education experience and attainment: Learning Chinese as a second/additional language in Hong Kong. *Teaching and Learning Chinese in Global Context,**5*, 63–80.

[CR119] Zhang, J., Li, H., Liu, Y., & Chen, Y. (2020). Orthographic facilitation in oral vocabulary learning: Effects of language backgrounds and orthographic type. *Reading and Writing,**33*(1), 187–206. 10.1007/s11145-019-09955-6

[CR120] Zhang, H., & Roberts, L. (2019). The role of phonological awareness and phonetic radical awareness in acquiring Chinese literacy skills in learners of Chinese as a second language. *System,**81*, 163–178.

[CR121] Zhang, L., Yin, L., & Treiman, R. (2017). Chinese children’s early knowledge about writing. *British Journal of Developmental Psychology,**35*(3), 349–358. 28025834 10.1111/bjdp.12171

[CR122] Zhao, A., Guo, Y., & Dynia, J. (2013). Foreign language reading anxiety: Chinese as a foreign language in the United States. *The Modern Language Journal,**97*(3), 764–778.

[CR140] Zhou, Y., & McBride, C. (2018). The same or different: An investigation of cognitive and metalinguistic correlates of Chinese word reading for native and non-native Chinese speaking children. *Bilingualism Language and Cognition,**21*(4), 765–781.

[CR123] Zou, Z., Zhao, W., & Li, M. (2022). The deficit profile of executive function in Chinese children with different types of reading difficulties. *Reading and Writing,**35*(3), 565–588. 10.1007/s11145-021-10194-x

[CR129] Zuber, S., Ihle, A., Loaiza, V. M., Schnitzspahn, K. M., Stahl, C., Phillips, L. H., & Kliegel, M. (2019). Explaining age differences in working memory: The role of updating, inhibition, and shifting. *Psychology & Neuroscience,**12*(2), 191.

